# Desert Related Death

**DOI:** 10.3390/ijerph182111272

**Published:** 2021-10-27

**Authors:** Mohammed Madadin, Rozanna Al-Abdulrahman, Shatha Alahmed, Rana Alabdulqader, Lama Alshehri, Norah Alkathery

**Affiliations:** Department of Pathology, College of Medicine, Imam Abdulrahman Bin Faisal University, Dammam 34212, Saudi Arabia; rozanna.alabdulrahman@gmail.com (R.A.-A.); Shathaalahmed1@gmail.com (S.A.); ranayaq@gmail.com (R.A.); Lama22sh@gmail.com (L.A.); N.j.36@hotmail.com (N.A.)

**Keywords:** desert, death, desert death, environmental, animal, medicolegal

## Abstract

Introduction: Desert death is defined as any death that occurs in the desert and could be attributed to a list of causes including environmental, animal related, undetermined, and other causes. Death in the desert seems to be obscure and little discussed in the field of forensic medicine, despite its importance, and there is only limited literature available on this broad topic. This narrative review aims to identify the most common causes of desert death and its medicolegal implications. Desert death causes: Environmental causes of death could be a result of temperature and lightening-related causes. Moreover, a variety of animals found in deserts are considered to be threatening and fatal, in addition to other and undetermined causes. Medicolegal implications of desert death: Likely to arise from the difficulties faced in finding the cause of death are the identification of the victim and the postmortem injuries that occur. Conclusion: Desert death is a broad topic with great medicolegal significance. More information and case reports need to be added in the literature. Guidelines for people about the danger of going to deserts in specific weather conditions should be implemented. Safety regulations must be taken into account at all times.

## 1. Introduction

Deserts and drylands are often referred to as terra nullius, barren spaces, and wastelands. However, they provide more than 2 billion people with dynamic biomes that cover more than 40 percent of the globe [1]. Deserts are described as regions of “low and untimely distributed rainfall, low humidity, high air temperatures, and strong wind”. Deserts are known to have a harsh environment; in some areas the summer temperature exceeds 48.8 °C and in winter the temperature falls below 0 °C [2]. The hot, hyper-arid and arid regions are called true deserts, and the desert fringes are semi-arid and dry sub-humid regions. Collectively, the world’s dry regions cover more land than any other significant form of climate [3]. The analysis shows that by raising a greater risk of extreme events, such as floods, heatwaves, hurricanes, and climate change, it is likely to have a direct effect on global health [4]. Over the past 50 years, climate change has caused deserts, already defined by climatic extremes, to warm and dry faster than any other ecoregions, leading to the decline or expiration of some species [5]. Numerous studies have indicated the effect of climate change on cardiovascular and respiratory mortality, but they were confined to specific regions, illustrating the fact that more research is required in this field, especially areas with a harsh desert climate [5,6]. A variety of environmental-related deaths has occurred in deserts, including hyperthermia, hypothermia, lighting, and envenomation. However, when faced with environmental death, reliable and detailed investigative information is of the utmost importance, as the cause of death can depend solely on circumstantial evidence, especially in the absence of substantial autopsy findings [7].

Calculating the number of deaths in the desert is difficult as there are no published statistics. In deserts, the deceased might be out of reach and missed, which will add to the problem [8]. Concerning immigration, as it is a major reason of finding people in deserts, there have been reports on the estimation of migrant deaths through the past years in the southwest United States-Mexico border [9]. From 1990 to approximately 1998, the Tucson sector in Arizona reported an average of 19 migrant deaths annually [10,11]. The number of deaths has increased regularly reaching 75 in 2001 in the Southwest United States border sectors [10]. In 2001, the United States established strict measures to control its borders, which led to a dramatic rise of migrant deaths in Arizona’s desert in 2005 [12]. From 1998 to 2013, more than 6500 migrant deaths have been reported. The highest score reported was in 2005, where the number of fatalities was approximately 492 [9,13]. The number of deaths continued to occur, nevertheless, there was a decrease in the number of apprehensions, which is a person who moves to live in a foreign country. In Africa, over 1790 deaths of migrants who moved from Sub-Saharan Africa to North Africa from 1996 and 2013 have been reported [9].

The Australian desert occupies over 70% of Australia, which consists of arid and semi-arid regions, and approximately less than 3% of the population live there. It is characterized by many difficult environmental factors that might contribute to death. For example, hot weather, unbalanced soils, and uncertain climate changes. Climate changes include high temperature, diminishing effective rainfall, and the occurrence of extreme climate events [14]. The recorded death rates in the Australian desert are markedly higher than the death rates in major cities in Australia by 10% to 70%. Aboriginal people were more affected than the non-Aboriginal population. Nevertheless, men living in remote areas are known to have higher suicide rates [14]. Desert death seems to be an uncommon subject as there is limited literature available on this topic. 

In this narrative review article, we aim to review common causes and medicolegal implications of desert death. 

## 2. Materials and Methods

To review the common causes and medicolegal implications of desert deaths, electronic databases have been used including Pubmed and Google scholar, beginning on 2 November 2020, by using search terms to locate traditional and online resources on this topic using searching strategy free text and MeSH. The descriptive search terms included *drylands, desert death, desert related deaths, desert death epidemiology, migrant death, prevalence of migrant death, environmental deaths, environmental related deaths, heatstroke, hyperthermia, hypothermia, lightning, wild animal attacks, fatal animal attacks, outdoor deaths, autopsy and artefacts, decay of human remains, border crossers, medicolegal, identification and postmortem*. No language restrictions were applied in the search results. Searching for titles was followed by abstract screening and then reading the full text of the related articles. This review included reviews that are related to desert deaths conducted worldwide and studies that were aiming to validate that cause of death was solely related to deserts and the environmental changes. Deaths in tropical climate areas and in mountainous areas were excluded. Moreover, migrants’ deaths outside the desert were excluded. Titles and abstracts were screened for eligibility. Relevant articles were included to develop this narrative review.

## 3. Desert Death Causes

There are multiple causes that can lead to death in deserts. According to the reported cases of deaths in deserts, we have classified the causes into four main categories, which are environmental, animal related, undetermined, and other causes of death.

### 3.1. Environmental

The direct impact of the high temperature weather on the human body is remarkable, including heatstroke, cramps, heat exhaustion, and death. However, the secondary impact has important consequences as well, such as the change in the susceptibility of diseases, infections, and change in the psychosocial determinant of health. An epidemiological study showed that less than 13% of deaths are due to heat effect [14,15].

#### 3.1.1. Temperature-Related Deaths

Hyperthermia is the most common cause of death in environmental-related deaths. It is defined as a body temperature over 38.3 °C [7]. Usually, death happens with higher temperatures above 40.6 °C [15]. As known, the decomposition is faster at high temperatures, so knowing the exact cause of death is a bit challenging. There are multiple causes accompanied by hyperthermia that have been found. For example, drug intoxication and ethanol intoxication [7]. However, several studies show that temperature-related deaths are higher in victims with cardiovascular and respiratory diseases [6]. Furthermore, some migrants in the Sahara Desert are kept in airless containers and trucks in extreme heat for a long period by smugglers [9]. On the other hand, many cases of unauthorized border crossers deaths in the Arizona Desert were due to exposure, hypothermia, or hyperthermia [16].

Heatstroke is a clinical emergency characterized by hyperthermia associated with delirium, seizure, and coma. It is fatal, could result in permanent nerve damage, and is considered to be a leading cause of desert deaths. People who are exposed to dry heat places like deserts are at a higher risk of having a heatstroke [17]. Hot climates like deserts will lead to dehydration, which will decrease sweat and predispose to heatstroke, and the expectation of death will result if a person loses more than 20% of total body water [18].

A study was conducted in China where they tested the effect of heatstroke on rats, and the results showed that the rats died at a temperature of 42.63 °C at the 170th minute with an increase to the maximum point of Heart Rate (HR) and Mean Arterial Pressure (MAP) then immediately decreased until the rats died. Firstly, HR and MAP were steady for a certain time, then fluctuated in a short term until the HR and MAP reached its maximum and was followed by a decrease, then death occurred [17]. Even though the environment is moist and hot, these symptoms take longer for a heatstroke to develop [19]. Other research shows that damage happens at a temperature greater than 42 °C in rats and humans [20,21]. Also, acidosis, congestion of blood vessels, and organ damage such as damage to heart, lungs, liver, and kidneys indicated by the changes of enzymology increased in severity with time. Moreover, a temperature of 41 °C is considered the start of serious heatstroke. The use of HR and MAP will also help in identifying the severity of heatstroke and monitoring the organ’s function by the aid of aspartate transaminase (AST), alanine transaminase (ALT), creatinine (Cr), urea, and uric acid. Under the microscope, the liver showed some changes, such as neutrophils in hepatic sinusoids, while the heart showed lymphocytes in capillaries between myocardial cells. The blood vessels to the vital organs are dilated, which leads to hypovolemic shock [17]. Besides, Multiple-Organ Dysfunction Syndrome (MODS) and Systemic Inflammatory Response Syndrome (SIRS) can appear in a dry heat environment [22,23].

Furthermore, fires in deserts could lead to death. This happens as a result of high temperature which may induce the spread of woody plants in some areas leading to hotter fires [14].

On the other hand, hypothermia is defined as a core temperature below 35 °C [7]. The cold weather at night could lead to sickness and death due to lack of medical treatment [9]. Deaths due to hypothermia usually occur as a result of ventricular fibrillation. Investigating a death due to hypothermia is not easy as it is a diagnosis of exclusion. Often, there is no specific finding but the presence of purple patches on the skin often over the extremities, Wischnewsky spots in the stomach mucosa, and pancreatic hemorrhage [7].

#### 3.1.2. Lightning

The least cause of death in deserts are caused by lightning. In the United States, approximately 100 to 600 deaths are due to lightning every year [24]. This happens when a high voltage direct current in the kiloampere (kA) range either strikes an individual directly, via a side-flash, or indirectly through an intermediary object. The indirect mechanism accounts for more cases of death than the direct mechanism. Animal rearers in developing countries are at a higher risk of lightning death. While Saudi Arabia is known for its semi-arid weather, except for in the southwest, with high temperatures at day and low temperatures at night, it has a low incidence of deaths caused by lightening [25].

In case of lightning, the cause of death is either cardiac or neurologic, cutaneous burns, or respiratory in origin. Many factors can play a role and affect the human body, for example, the intensity of the current, the timing of its passing through the body, the pathway involved, the activity and position of the person at the time of the event in relation to the ground, in addition to the mechanism of striking [24,25].

There are different mechanisms affecting the body. First, the direct strike which is considered the most damaging to the body. It enters the body through the head or outstretched arms and exits through the soles because the body is usually standing. Second, the contact voltage occurs when the person is holding a metal object such as a phone or weapon. The lighting hits the metal object then the current flows to the body. Third, side flash happens when the lighting strikes a near object first then the current travels down to nearby people. Last, the ground strike, which happens when lighting strikes the ground and the person is walking or standing nearby. Usually, the heart and brain are spared since the voltage passes through the lower extremities [24,25].

The findings rely on the scene, where the body will be outside after a storm with torn clothes, burned with cutaneous thermal injuries, singed body hair, and ruptured tympanic membranes. Five cases were found in the Sonoran Desert from July 2010 to July 2015. One case reported a lightning-related death in a desert in the Northern Border Province, Saudi Arabia. The body of a 50-year-old male was discovered with wet, burnt, and torn clothes with multiple different degrees of burns, erythema, some erosions, and singed hair in different parts of the body, distributed in a cranio-caudal direction. Erythema is mostly caused by steam burn that formed by the steaming of the rainwater. Autopsy and histopathology reports showed an edematous lung with ruptured peripheral alveoli and pneumothorax, surgical emphysema, and pneumo-mediastinum. The death was mostly due to electrical disruption of the cardiac cycle [6].

### 3.2. Animal Related

Wild animals and even domesticated animal attacks can cause severe injuries and death by various mechanisms. For instance, sharp and blunt trauma, crush asphyxia, vascular injuries with hemorrhage, internal organ injuries, envenomation, poisoning, sepsis, and anaphylaxis [26].

There are 25,000 deaths reported annually due to venomous snakebites [27]. Venom poisoning happens as a result of anaphylaxis or toxic reaction. Snakebites poisoning is more common than toxic reactions from a variety of insects or arthropods by stings or bites. For example, there was a reported case in the Sonoran Desert where the victim was killed by an Africanized honeybee, known as the killer bee, which is found all over the desert [7].

Snakebites are one of the important causes of desert deaths. In a case reported in the Sonoran Desert, a female was bitten by a venomous snake. However, due to the decomposition of the body, the area of the bite could not be seen, but the person that accompanied her confirmed that it was a venomous snake. Death was certified as undetermined and the snakebite and hyperthermia due to exposure were considered to be contributing conditions [7]. Furthermore, an observational facility-based study conducted in Thar Desert, Pakistan from November 1995 to October 1996 reported four cases of death due to snakebites among 771 victims. Usually, snakes become more active in hot weather and most of the biting injuries occur after sunset since the darkness limits their vision [27].

Another type of animal that is highly susceptible to cause death in the desert is the scorpion. Worldwide, more than 32,000 deaths due to scorpion bites have been reported annually. Approximately 2000 types of scorpions have been described globally. The fatal scorpions’ habitat is commonly in warm areas. For example, *Odontobuthusdoriae,* which is classified as Diggers, usually live in deserts and non-residual regions. Another fatal scorpion is called *Androctonuscrassicauda*, and can be found in ground holes and splits, under stones, and inside wild animals’ dens. It is characterized by thick skin which protects it from the difficult habitat, and it can exist in various areas. Furthermore, *Hemiscorpuslepturus* is the most dangerous scorpion, although it has thin skin which limits its spreading in dry hot deserts. However, there is not enough data about desert deaths due to scorpions in particular, but it can be concluded that people in deserts are at a high risk of fatal scorpion bites [28].

Many fatalities were caused by insects such as spiders, dogs, pigs, cattle, horses, and even buffalos [26,29,30]. Wild pigs typically are not fatal, even though, there are four reported fatalities due to wild pig attacks on humans in the US [29]. The pig may attack a human by crushing trauma and biting [29,30]. Likewise, there are several reported deaths due to camel attacks [26]. Camels are characterized by their unique ability to tolerate difficult environmental circumstances in the desert. For example, hunger and thirst for many days and an arid, hot climate, which make the desert the best area for rearing camels. Usually, camels are used as a source of meat and milk or for riding (such as their use for transportation). Camels might be dangerous and kill humans in some situations, for example, when the male has been taken away from the female [31]. Camels attack humans by kicking their head, chest, or abdomen. Moreover, they may trample on and crush victims leading to fractures and serious injuries. Another traumatic mechanism is falling off or being thrown off the camel. Camel bites could be very dangerous as they may injure big arteries. Additionally, a vehicle collision with a camel may lead to death [26,32]. 

Wild elephants can also be considered as fatal animals. In India, approximately 300 fatalities every year are due to wild elephant attacks. It was noticed that the majority of attacks were sudden and unexpected and that the elephants were targeting the chest and the head to crush and trample on [33].

Generally, fatal big wild cat attacks on humans are rare. Between 2003 and 2012, an average of three individuals were fatally injured due to wild pig attacks [34]. The leopard, which falls under the feline family and exists in the wild, is described as the most vigorous feline in the world after the jaguar. A leopard can pull a victim who is heavier in weight up a tree. Its canines are extremely strong which deeply penetrate the victim’s tissue. There is a reported case of a leopard attack in Africa, otherwise, fatal feline attacks on humans are very rare worldwide. Leopards mostly target the neck vessels and neck airway when they attack their victims [35]. Correspondingly, the lion is the second biggest wild cat in the wildlife. It is known that female lions have the best skills for hunting. In fact, male lions are very skillful and usually prefer to hunt at night. One case reported a sudden unexpected attack of a lion on a human in Brno City, Czech Republic in 1973 [34].

### 3.3. Other Causes of Deaths

The desert itself could be the major cause of death [36]. For instance, in the Sahara Desert, death can occur due to smugglers, traffickers, and banditry. Sometimes, due to lack of preparedness for food, such as low nutrient foods and water, which can lead to thirst, starvation, and exhaustion. Nevertheless, getting stuck in deep mud is considered to be another environmental danger [9].

Migrants constitute a large proportion of the reported cases of desert deaths. The surviving migrants have reported that they found a large number of dead bodies as a result of neglect and murder by torture, gunfire, or even victims of organ removal in the desert as they were traveling [9].

U.S.-Mexico unauthorized border crossers face a lot of fatal circumstances including heatstroke, dehydration, snake, automobile accidents, snakebites, getting lost, criminal gangs’ prey, raping, robbing, and direct violence [2]. Assailants and kidnappers threaten to steal their money, and the Border Patrol agents who look for their records are all considered to be fatal dangers for migrants [37]. Crossers who are unprepared for the desert terrain usually lack basic water and food to survive [2].

To stop migration, the U.S. Border Patrol budget and personnel had been doubled, a security wall, and other technologies had been applied. This led to create a “funnel effect” that caused an increase in crossers in remote areas and more migrant deaths [2].

### 3.4. Undetermined

In case the deceased was identified by only some of the skeletal remains, the cause of death is reported as undetermined [10]. This is the result of the body being too decomposed to identify the exact cause, for example [7].

## 4. Medicolegal Implications of Desert Deaths

### 4.1. Difficulties in Finding the Cause of Death

Identifying the cause of death in such cases is very challenging and may even turn out to be impossible. In some cases, the desert may turn out to be a secondary scene. For example, in cases of homicide, the body of victims may be moved from the actual crime scene to the desert where the body was retrieved. Other examples include when victims die for any other cause, such as death due to drugs, alcohol, or even natural disease. The factors causing difficulty in identification and the postmortem injuries caused by animals contribute to making the determination of the cause of death more difficult [38].

### 4.2. Difficulties Faced in Identification

Identifying victims who die in deserts remains a great challenge for medical examiners. Many obvious reasons may be mentioned but dying in a remote area will most definitely be at the top of the list [38]. Sometimes, corpses are recovered due to migrants, hikers, humanitarian workers, or researchers that report to law enforcement [8]. The dead body of a victim left alone in the middle of nowhere experiences many changes that render difficulty in the process of identification [38].

Scavenging of the victim’s body by animals and human trekkers play a role in reducing the available evidence that may lead to the identification of the victim. [38]. In addition, human remains may be found in a fragmented state due to their destruction by animals and environmental conditions. Animal scavenging in particular may render the recovery of victims impossible [8]. Clothing items may be destructed, paper and plastic items such as identification documents may turn out to be unreadable, and DNA may be difficult to extract. This may all be due to the effects of weathering, particularly intense and protracted sunlight. These destructive forces will not spare the human body of course. Tattoos, scars, and others can be difficult to see within a day or even less as a result of hot and dry weather [38].

Body cooling may be used as an indicator for postmortem interval in the first 24 h after death [38]. However, this method may not be reliable in extreme climates as in this case, desert regions may cause the body temperature to rise after death [39,40]. During the decomposition process, the anterior dentition falls from the alveolus. This could be aided by animal scavenging as they are likely to jostle the skull. Teeth are considered to be important identifiers as they are most likely to be recognized by the people who might know the victim. As the exposure increases, the chance of identification decreases as well [38]. A fresh appearance of the remains of a victim may be retained for a considerable time. However, during summer months, marked decomposition occurs rapidly [39]. A year or more of exposure of bones and teeth to sunlight may cause cracks. Consequently, they may lose the potential of identification due to loss of surface detail. In the absence of antemortem, dental records, and fingerprints, help from families may be more reliable [38].

Another important post-mortem change worth to be mentioned is the process of mummification. This process requires special circumstances to occur. Mummification is considered to be a modification of putrefaction that occurs in dry environments and low humidity such as deserts [39,40,41,42]. Moreover, it can be facilitated under conditions of extreme heat and cold. This process happens as a consequence of the evaporation of body fluids [42]. The underlying importance of mummification is that it preserves tissues which dramatically increases the likelihood of the identification process and injury recognition [40,41]. To some extent, it may give an idea about the postmortem interval. However, the precise time required for the completion of mummification usually may not be stated [40]. It is wise to note that although mummification preserves the tissues, rodents and insects may attack the mummified tissues [41]. A common insect that is usually found in many countries worldwide is the omnivorous larvae of the brown house moth which is also known as Hofmannophila pseudospretella [40]. 

Antemortem issues faced are also considered as significant obstacles. Taking the migrants that illegally cross the United States (US) international borders with Mexico through the Sonoran Desert as an example, many times their families are unable to come to the US in order to help in the process of identifying the victim. When possible, routine digital photographs of the face, personal items, clothes, and anterior dentition taken by the staff members are shown to the family, mostly after the postmortem examination [38]. It is wise to ask all family members involved in visual identification and to provide tissue samples and antemortem data, as they may potentially lead to the confirmation of the victim’s identity [43]. Moreover, on some occasions, participation in the process of identification is refused by the family. This is most likely due to their fear of the probability of facing legal charges, or because of the fear of confronting the agonizing pain of their loss. Furthermore, most of them have limited access to the internet, telephones, and fax machines. Consequently, an interpreter should help in communicating postmortem descriptions and findings. As a result, this delay possibly inhibits identification of the victim. In the event of uncooperative families, identifying the victim may be impossible as their help is of vital importance [38].

Sometimes, after the examination of personal items, addresses and telephone numbers may be found which may lead to an identification [38]. However, in the case of migrants for example, if any documents are found it is important to note that false documents are carried by many of them [43].

### 4.3. Postmortem Injuries

Outdoor corpses are more prone to animal scavenging as their main motivator is hunger and the corpses are defenseless. Natural putrefaction of the corpse releases odors that in turn attracts the attention of wild animals. This leads to the complete destruction of corpses and scattering of the victims’ bones and belongings over a very large area [44]. Discriminating lesions inflicted by animals from trauma in the antemortem and postmortem phase and their contribution to the cause of death may cause significant difficulties during the autopsy [44,45]. In addition, with the occurrence of significant destructive changes to the corpse, for example, the face and teeth, many issues may rise concerning forensic anthropology and forensic pathology. Furthermore, when the attacking animals devour the internal organs of the victim, identification of the exact cause of death may be impossible, even if the victim has died due to natural consequences. This also happens in cases where larvae completely cover the corpse as they may erase tattoos and surgical scars which are considered to be diagnostic cutaneous marks [44].

Open wounds caused by posttraumatic death are usually the immediate first target for scavenging animals. Consequently, the identification of antemortem wounds turns out to be very difficult. A variety in the sizes of the animals dentition and jaws leads to a variety in the lesion extent. However, they do share some common characteristics, such as lack of significant bleeding from the bitten tissue, absence of active bleeding, edema, and erythema on the wounds’ edges [44]. Many mechanisms of animal attacks may cause death. These mechanisms may involve blunt trauma, sharp trauma, crushing, envenomation, anaphylaxis, and sepsis. Sometimes these mechanisms may render difficulty in determining the cause of death and may deceive the examiner into believing that the cause of death was caused by another mechanism [44,45,46]. These lesions may be misinterpreted as antemortem lesions such as firearm wounds, stab wounds, or even ligature marks [44].

Blunt traumas caused by animal attacks may be confused with homicides. These attacks may be witnessed or finding some clues may confirm that the injury was truly caused by animals. However, in the absence of evidence difficulties may arise in finding the actual cause of injury. This means that excluding human activity requires extensive and careful scene evaluation. Taking envenomation as an example, bite marks may not be obvious in snake bite victims during the autopsy. Moreover, the absence of stings in anaphylactic deaths does not exclude bee or wasp attacks. In this case, ancillary studies including serum tryptase and specific IgE levels should be undertaken. Mild animal bites may lead to unusual infections in immunocompromised victims. Some lesions caused by animal attacks present on hands may be misinterpreted with defense wounds [41]. Suspicions may be raised in cases were clothes of the victim are open, genital organs are exposed, and trauma to the genitalia or anogenital region are present, as it may seem like a sadistic sexual assault and homicide with mutilation [44,46].

A wide variety of birds prefer feeding on the eyes of corpses. Investigators may believe that this is a sadistically committed crime or even a ritual mutilation that has been performed on corpses. They may also leave wounds that seem like surgical cuts and teeth marks on the center of long bones and flat irregular bones. The wide variety of beak morphology causes different lesions. Other examples include the formation of holes as a result of worm activity which may be confused with holes caused by bullets. They may also cause skin defects which may resemble penetrating stab wounds. Worms feeding on the victim may aid in identifying the victim by DNA. This is accomplished by analyzing the worm’s gastrointestinal contents [44].

Superficial, serpiginous, parchmentized, and irregular shaped skin lesions are generally formed from feeding by ants. They also cause scattered orange-pink wound marks on the surface of the skin and characteristically remove the epidermal layer. Small punctuate and scratch lesions caused by ants may be deceiving as they may seem like antemortem abrasions and acid wounds. Moreover, when the corpse remains in the same position for a long time, some regions of the body congest. As a result, ant bites at the terminal end of the dermal capillaries may bleed extensively. This may be misinterpreted and may resemble as trauma. Furthermore, lesions localized on the neck may be misinterpreted as hanging marks. One of the findings that may show an association of ants is the absence of eyelashes, as ants characteristically remove them [44].

Other insects such as cockroaches may cause lesions that resemble skin disease as they inflict superficial dermal abrasions. The activity of carnivorous animals may be falsely interpreted as stab wounds just as those caused by knives. This is the result of their canine teeth and the linear scratches inflicted by their paws [44].

Postmortem injuries caused by animal attacks are mostly incidental. However, feeding the remains of victims to animals by the criminal is a way to attempt concealing the homicide. In addition, the establishment of the cause of death may not be possible in cases where there is removal of tissues. Furthermore, it may not be possible to determine whether the injury contributed to death or was followed after death [46].

Knowing the location of the victim, animal species in that area, season of the year, and geographic conditions is very helpful in determining the animal that caused the attack. Furthermore, the behavior of the animals in that specific location and the types of animal feeding may further help in identifying the animal. In rare instances, deaths due to poisoning may lead to death of the scavenging animal due to the animal feeding on the poisoned victim. These animals may be found at the incident site [44,47].

In order to distinguish if the animal attack occurred during the antemortem or postmortem period, many histological, histochemical, and immunohistochemical methods can be utilized. Histological examination may show changes in the wound with time and the cell types that function in wound healing. Moreover, histochemical examinations enable the analysis of fibroblastic enzymes. However, examiners should note that the reliability of these tests are low. Furthermore, adhesion molecules, inflammatory cytokines, and transforming growth factors (TGF) may be analyzed by immunohistochemical methods to discriminate between antemortem and postmortem lesions [44].

## 5. Conclusions

It is important to mention that the reported cases of desert deaths and desert-related deaths are few. More information and case reports need to be added in the literature. Understanding specific circumstances of desert deaths might help forensic pathologists and authorities in concluding the cause and circumstances of death.

In order to decrease the incidence of desert deaths, guidelines for people about the danger of going to deserts in specific weather conditions should be implemented. Safety regulations must be taken into account at all times. Preserving plenty of water is very crucial to prevent dehydration and replace that lost from sweating. Eating snacks is important for replacing electrolytes that are lost from excessive sweating. Never approach nor feed any wild animal. Pay attention to the many venomous animals that live in burrows. Consider car and road preparations e.g., full fuel tank, avoid off-road driving. Advise people never to hike alone and make sure to check the weather forecast beforehand. When visiting the desert in winter, extra clothing is important to avoid hypothermia and always carry a first aid kit [48].
